# Case Report: Hypergonadotropic hypogonadism in an elite athlete with variant in FSH receptor gene—clinical and sports implications of proper diagnosis

**DOI:** 10.3389/fendo.2025.1648940

**Published:** 2025-08-22

**Authors:** Monika Skrzypiec-Spring, Agnieszka Zubkiewicz-Kucharska, Magdalena Pasińska, Michał Rabijewski, Robert Śmigiel, Adam Szeląg

**Affiliations:** ^1^ Department of Pharmacology, Wrocław Medical University, Wrocław, Poland; ^2^ Department of Pediatrics, Endocrinology, Diabetology and Metabolic Diseases, Wrocław Medical University, Wrocław, Poland; ^3^ Department of Clinical Genetics, Ludwik Rydygier Collegium Medicum in Bydgoszcz Nicolaus Copernicus University in Toruń, Bydgoszcz, Poland; ^4^ Department of Reproductive Health, Centre for Postgraduate Medical Education, Warsaw, Poland

**Keywords:** hypergonadotropic hypogonadism, FSHR gene mutation, infertility, c.2039A>G variant, FSH treatment

## Abstract

Hypergonadotropic hypogonadism is usually caused by the impairment of the structure and function of the gonads, but there are cases caused by reduced stimulation by the follicle-stimulating hormone (FSH) as a result of abnormal variants of genes encoding the follicle-stimulating hormone or its receptor (FSHR). We present the case of an elite athlete with the c.2039A>G variant in one allele of the FSHR gene resulting in hypergonadotropic hypogonadism, low testicular volume, and reduced semen parameters, placing particular emphasis on the diagnostic process and the importance of correct diagnosis in the context of possible treatment with gonadotropins, which can significantly improve fertility, increase testosterone levels, and, in the case of athletes, obtain approval from the anti-doping organization for treatment that increases testosterone levels.

## Background

Hypergonadotropic hypogonadism is usually caused by the impairment of the gonads with a normally functioning hypothalamic–pituitary system. However, there are cases of hypergonadotropic hypogonadism evoked by reduced stimulation by the follicle-stimulating hormone (FSH). FSH acts via FSH receptor (FSHR). The FSH receptor (FSHR) is expressed in the granulosa cells of the ovary and in the Sertoli cells of the testes. Its activation promotes folliculogenesis and supports spermatogenesis in cooperation with the activated luteinizing hormone (LH) receptor (1).

The FSHR gene is located on chromosome 2p21–p16, and it is a single copy gene being 54 bK long. There are multiple isoforms and numerous variants, such as single nucleotide polymorphisms (SNPs) of FSHR. Approximately 1800 SNPs of the FSHR gene in the SNP database have been reported (2, 3). These structural changes may affect the receptor functional properties, thus enhancing (gain-of-function mutations) or impairing (loss-of-function mutations) the receptor activity. Inactivating mutations reduce the receptor’s function up to a total block, altering either the formation of the receptor–ligand complex or FSH signal transduction. In women, inactivating mutations of the FSHR are causing amenorrhea, both primary and secondary; premature ovarian failure (POF); and infertility. Total inactivity of FSHR results in a block of follicular maturation and is associated with abundant small follicles as in prepubertal ovaries (4, 5).

In men, the clinical picture associated with FSHR inactivation is heterogeneous: some cases presented in the literature show a normal pubertal development, with discretely decreased testicular volume, level of testosterone within normal ranges, normal or moderately elevated LH, and high FSH. They show with variable spermatogenic failure, from severely and moderately reduced sperm counts or low volumes of sperm fluid with normal sperm concentrations. On the other hand, there are also patients with small testes and azoospermia. Testosterone levels varied from low up to normal. The reason for this discrepancy remains unclear; however, a persistence of a residual function of FSHR in some genetic variants cannot be excluded (4, 5).

The FSHR 2039A>G variant is associated with decreased receptor sensitivity due to an asparagine-to-serine switch in the FSHR intracellular domain (6). The clinical manifestation of the FSHR 2039A>G variant may include increased FSH levels, low inhibin B levels, impaired spermatogenesis, small testicles and low testosterone (T) levels, and high LH levels. In men with the FSHR pathogenic variant, FSH treatment resulted in improved semen quality (7).

We present here the case of an elite athlete with hypergonadotropic hypogonadism caused by an FSHR causing-disease variant.

## Case presentation

A 17-year-old elite athlete presented to an endocrinologist and sport medicine specialist in April 2024 to determine the course of action in connection with his increased FSH and LH levels. Diagnostics began in December 2023 due to unilateral enlargement of the mammary gland.

Gynecomastia was observed for the first time a year ago, together with weakness and deterioration of concentration. The patient did not notice any other signs or symptoms; he continued his regular professional training routine.

He denied taking stimulants and supplements/nutritional supplements of unknown origin. His medical history was not relevant; the boy did not recall any testicular injury or severe pain but stated that he has felt discomfort in the testicles several times. He was vaccinated in accordance with the National Vaccination Program, including the mumps–measles–rubella (MMR) vaccination.

At the time of presentation, the patient’s body height was 183.0 cm; body weight was 85 kg. The patient had a proportional and athletic body build; the physical examination was unremarkable. Puberty status corresponded to grade V on the Tunner scale. However, a small testicular volume was found of approximately 12 ml with a varicocele on the left side. Additionally, the patient had unilateral enlargement of the mammary gland with palpable glandular tissue, single skin striae.

Laboratory test results allowed suspicion of hypergonadotropic hypogonadism and revealed a slightly increased prolactin concentration (**Table 1**). In order to confirm those diagnoses, a GnRH test, as well as a metoclopramide (MCT) test and prolactin profile, was performed. In the GnRH test, a 10-fold increase in LH concentration and a two-fold increase in FSH concentration were obtained, which allowed to confirm the diagnosis of hypergonadotropic hypogonadism; the metoclopramide test done to diagnose hyperprolactinemia revealed a 3-fold increase in prolactin concentration in the 60th minute of the test. Moreover, the daily prolactin profile was rigid. In the following months, the concentrations of gonadotropin and, prolactin remained at a similar level (**Table 1**). The results of other hormonal tests (including adrenal hormones – cortisol, DHEAS, androstenedione and 17(OH)progesterone, as well as thyroid function – TSH, fT4 and fT3) were within the normal ranges during the entire observation.

**Table 1:** The concentration of selected hormones during the observation.

	Normal range	Initial results	+2 months	+4 months	+6 months	+12 months	+15 months	+18 months
LH [mIU/ml]	1.7–8.6	7.9	9.6	9.48	5.46	7.41	0.439	0.887
FSH [mIU/ml]	1.5–12.5	29.8	27.5	28.42	29.4	32	2.69	9.72
Testosterone [ng/ml]	1.88–8.82	4.50	3.57	3.66	4.8	2.17	3.12	4.03
Estradiol [pg/ml]	11.3–43.2	32.9	NA	23.36	NA	<20.0	<20.0	<20.0
Prolactin [ng/ml]	4.0–15.2	26.6	29.1	28.21	22.9	21.0	25.6	26.3

NA, not applicable.

Resting testosterone concentration was in the lower limit of the normal range, with a decrease observed in the following months (**Table 1**). The chorionic gonadotropin (hCG) test was used to assess Leydig cell function, and a significant increase in the concentration of this hormone was observed after hCG stimulation (**Table 2**). Estradiol concentration was within higher limits of the normal range throughout the entire observation. Inhibin B was below lower limit of the normal range (<10 ng/ml on +6 months and 27 ng/ml on +12 months; normal range 120–400), Anti-Müllerian hormone concentration was within normal range (2.60 ng/ml on +6 months and 2.61 ng/ml on +12 months; normal range 1.43–11.60).

**Table 2:** Stimulation test results done during hospitalization (+6 months of follow-up).

GnRH test	0’	30’ after GnRH		60’ after GnRH	120’ after GnRH	24 hours after GnRH	96 hours after GnRH	
LH [mIU/ml]	5.46	48.3		50.8	40.3	60.9	1.5	
FSH [mIU/ml]	29.4	51.2		57.3	62.6	117.0	16.9	
hCG test	0’			24 hours after hCG	48 hours after hCG		72 hours after hCG	
Testosterone [ng/ml] (normal range 1.88–8.82)	5.17			8.88	7.38		10.2	
Estradiol [pg/ml] (normal range 11.3–43.2)	79.5			91.2	44.4		76.4	
Metoclopramide test	0’			60’ after MTC			120’ after MTC	
Prolactin [ng/ml] (normal range: 4.0–15.2)	21.75			60.45			44.80	
Prolactin profile	8:00	11:00	14:00	17:00	20:00	23:00	2:00	5:00
Prolactin [ng/ml] (normal range: 4.0–15.2)	26.3	22.9	23.2	23.9	19.2	27.8	25.3	26.2

Due to one time that a high concentration of cortisol was demonstrated in the evening (264.8 nmol/L, 9.6 μg/dl), it was decided that a low-dose dexamethasone suppression test be performed—the result of this test (cortisol level at 8:00 of 38.6 nmol/L, 1.4 μg/dl) showed proper suppression of the cortisol secretion (<50 nmol/L, <1.8 μg/dl), and the morning ACTH concentrations (8:00—27.1 pg/ml, 24.9 pg/ml, normal range <46 pg/ml) were also within the normal ranges. In addition, a discreetly increased concentration of beta-hCG was found (1.9 mIU/ml, normal range <1.1). Hemochromatosis as a possible cause of hypogonadism was excluded.

The ultrasound examination performed initially showed normal echogenicity and echo structure of the testicles, with unchanged vascularity; the given testicular volume was calculated as 8.5 ml. The testicular ultrasound examination performed after three months showed a decrease in testicle volume of up to 6.5 ml (right) and 4.2 ml (left), as well as varicocele of the left spermatic cord of up to 4.3 ml. Echostructure and vascular flow through the testicles were preserved; testicle sizes were 6 ml (right) and 5.3 ml (left), and there was varicocele on the left side.

A breast ultrasound was performed, which confirmed double-sided gynecomastia.

Additionally, in the initial phase of diagnosis, the semen analysis revealed 2.4 ml of sperm per ml of semen and oligoteratozoospermia. Semen cultures for aerobic and anaerobic bacteria as well as for fungi were all negative. Tests for chlamydia and *Ureaplasma* yielded negative results. Furthermore, antibodies against spermatozoa IIF (ASA) and antibodies against Leydig cells were all negative. The boy underwent sperm cryoprecipitation.

In order to differentially diagnose hyperprolactinemia, an MRI scan of the hypothalamic–pituitary region was performed, revealing a small zone of weaker contrast saturation, measuring 2.5 × 1.8mm parasagittally on the right side, which could suggest the diagnosis of pituitary adenoma. As mentioned before, hypothyroidism, which may result in a slightly increased prolactin level, has been ruled out. The degree of increase in prolactin concentration in the metoclopramide test, as well as the result of the daily prolactin profile allowed the exclusion of a pituitary adenoma of the *prolactinoma* type. The control MRI scan of the hypothalamic–pituitary region, performed after approximately 9 months, ruled out the presence of the previously described lesion. All these indicated that the focal lesion described in the initial MRI examination was hormonally inactive (incidentaloma), and the mild hyperprolactinemia could have been the result of chronic stress in the patient or was an artifact. The functional nature of the hyperprolactinemia was also supported by the fact that during the patient’s post-hospital follow-up, one of the prolactin levels was normal.

Series of genetic tests were conducted: the Klinefelter syndrome was excluded, as a normal male karyotype was found, 46 XY, including karyotype testing from skin fibroblasts; also, the FISH test confirmed the correct distribution of signals for sex chromosomes, without loss of a fragment of the Y chromosome in the SRY region, and no numerical abnormalities of sex chromosomes were found. The patient underwent molecular testing of the FSHB and FSHR genes, which revealed the c.2039A>G variant in one allele in the FSHR gene and the absence of mutations in the FSHB gene.

The testosterone supplementation treatment was recommended; however, initially, in order to stimulate spermatogenesis to prepare better material for sperm cryoprecipitation, the patient received follitropin recombinant (75 IU three times a week) and hCG recombinant (initially 1000 IU three times a week, later reduced to 900 IU three times a week). In a follow-up ultrasound examination performed 3 months after the end of treatment, the volume of the left testicle increased to 5.5 ml from its initial value of 4.2 ml, or by 30.9%. Up to now (6 months of treatment, 18 months of observation), the therapy resulted in an increase of testosterone concentration, as well as normalization of gonadotropins levels. The testicular volume, however, remained unchanged. Unfortunately, the post-treatment semen analysis was not yet performed because the patient was unable to collect a semen sample for analysis. The patient did not report any physical complaints but still required psychological care.

The diagnosis of hypogonadism, reduced testicular volume, and impaired fertility in a 17-year-old teenager, as well as the diagnostic process itself, which concerned a very intimate area of one’s life, had an enormous negative impact on the patient’s psyche and mood.

The patient received significant support from his family and remained under the constant psychological care of his sport psychologist. Therefore, during the diagnosis and treatment, we did not interfere with the psychological therapy he received but merely monitored whether it was being implemented and whether it was effective.

At the beginning of the diagnostic process, the patient and his family were provided with a detailed explanation of the possible underlying conditions responsible for his symptoms, the subsequent diagnostic steps, the prognosis depending on the final diagnosis, and the therapeutic options. Increasing the patient’s awareness and providing a detailed explanation of the diagnostic process contributed to the patient’s patience and understanding and significantly reduced his anxiety. Cryoprecipitation of semen at the beginning of the diagnostic process also had a greatly beneficial effect on the patient’s emotional condition, as it gave him the confidence that in the absence of effective treatment, his fertility was protected.

The patient and his family repeatedly emphasized that the diagnosis and initiation of treatment were of fundamental importance, as they brought them relief. Thanks to the improved results, his mental state and quality of life improved. The boy has returned to a normal lifestyle, including intensive sports training and competition.

## Discussion

A possible role of SNPs of FSHR on reproductive functions in men is still controversial. FSHR 2039A>G SNP is suggested to decrease the receptor sensitivity and subsequent elevation of FSH level, decrease inhibin B levels, and decrease testicular volume in men (8–10). An association between FSHR SNPs with sperm concentration and total sperm count was also found (6). Several studies have found an association between SNPs (including FSHR 2039A>G) and lower testosterone levels and higher LH levels or lower calculated free T/LH ratio, indicating an effect on the function of Leydig cells (8–11).

The clinical manifestation of FSHR 2039A>G SNP may therefore include increased FSH levels, low inhibin B levels, impaired spermatogenesis, small testicles and low T levels, and high LH levels, although the clinical picture may vary, among other things, due to the differences between homo- and heterozygous variants. Data in the literature regarding the differences between heterozygous and homozygous FSHR variants are sparse. The main point emphasized is that the homozygous variant has been shown to be associated with significantly higher basal serum FSH levels. There are no clear data regarding what other differences in clinical presentation occur between heterozygous and homozygous FSHR variants and what the differences in response to treatment are (5, 6).

In the case described, the first step was semen analysis. Live sperm was obtained from the patient and cryoprecipitated to protect the patient’s fertility. Such treatment should be given priority when low testicular volume and significantly elevated FSH values are detected and should be performed before extending the diagnostics, as its postponement may reduce the chances of obtaining sperm that can be subjected to cryoprecipitation.

Then, in order to make therapeutic decisions aimed at improving spermatogenesis and obtaining normal testosterone concentrations and in order to obtain a Therapeutic Use Exemption (TUE) for this treatment from the Polish Anti-Doping Agency (POLADA), the patient underwent detailed diagnostics. A TUE allows athletes to use substances on the Prohibited List of Substances and Methods in sport if they have a disease or condition that requires the use of specific medication. Drugs that increase testosterone levels and testosterone itself are on this list. First, Klinefelter’s syndrome and infections in the urogenital system were excluded. After obtaining negative results for Klinefelter syndrome and secondary testicular damage, the overall clinical picture could indicate gonadal dysgenesis; however, to confirm this diagnosis, autoimmune causes of testicular damage and gonadotropin dysfunctions had to be (and were) excluded. Additionally, the presence of a suspicious lesion, presumably apituitary adenoma, required in-depth endocrine diagnostics regarding its potential secretory activity in the field of gonadotropins, prolactin, and ACTH. At that time, the patient was referred to the pediatric endocrinology department. There, the presence of antisperm antibodies was excluded, as well as pituitary secretory overactivity in the field of prolactin and ACTH. A gonadotropin stimulation test (GnRH test) and human chorionic gonadotropin (hCG) stimulation tests were performed in order to assess the pituitary’s ability to secrete gonadotropins and to assess Leydig cell function. The results of these tests revealed no increase in testosterone concentration under the influence of endogenous gonadotropins and a strong response to exogenous hCG together with a decrease of gonadotropin levels. The results of these tests argued against the secretory activity of the pituitary adenoma in terms of gonadotropin secretion and for an abnormality in the action of endogenous gonadotropins, which was later confirmed by the genetic test result revealing the mutation in the FSHR gene. Due to the good clinical response to exogenous hCG and the results of previous studies indicating the improvement of spermatogenesis under the influence of exogenous FSH in patients with FSHR SNP (10), a decision was made to initiate human menopausal gonadotropin (hMG) and hCG treatment in order to improve spermatogenesis and cryoprecipitation of semen with better parameters than the semen samples obtained before treatment, alternating with testosterone replacement treatment. The applied therapy resulted in enlargement of the testicular volume and normalization of gonadotropins and testosterone levels.

The effectiveness of treatment with an exogenous FSH receptor ligand has been clinically confirmed in several studies, although the mechanism responsible has not been explained (7, 12, 13). Similarly to the lack of a clear explanation for the effectiveness of an exogenous FSH receptor ligand with reduced sensitivity to the endogenous ligand, we do not know a clear explanation why hCG stimulation led to an increase in testosterone levels with reduced sensitivity to the endogenous ligand in this case. Based on the results of experimental studies, the effects of Sertoli cell dysfunction on Leydig cell dysfunction are postulated probably due to impaired paracrine communication between nuclear compartments, but the factors controlling this effect remain incompletely understood (14). A number of proteins are potential paracrine regulators of Leydig cell function. One of them is IGF-1, which is expressed in the seminiferous tubules of adults, and its receptor is expressed in Leydig cells (15, 16). IGF-1 is known to regulate testosterone production. FSH receptor antiserum has been shown to lead to a reduction in StAR and IGF-1 mRNA levels (17). In turn, StAR is essential in the acute response to LH signaling in Leydig cells in the initial phase of testosterone production triggering cholesterol movement from the outer mitochondrial membrane to the inner mitochondrial membrane (18). In the described case, the reduced testosterone concentration may have resulted from the existence of such a relationship between Sertoli and Leydig cell dysfunction, and this may be related to the response to exogenous hCG in the absence of response to the endogenous ligand.

In the presented case, the patient had a heterozygous FSHR variant, which theoretically could be associated with a good and long-lasting response to treatment. However, to our knowledge, the described case is the first reported evidence that gonadotropin therapy in FSHR variants can lead to permanent restoration of testicular hormone production, so there are no data on whether there are differences in treatment response between heterozygous and homozygous FSHR variants.

Conducting detailed diagnostics allowed this patient to be treated with the promise of obtaining sperms with better parameters than those obtained before treatment and increasing his chance of maintaining fertility. Additionally, demonstrating the cause of hypogonadism allowed him to obtain an exemption for therapeutic purposes, which, in the case of drugs that increase testosterone levels, can only be obtained after clearly demonstrating the irreversible cause of hypogonadism, and allowed him to continue his professional sports career.

To summarize, incorrect and incomplete diagnosis of hypergonadotropic hypogonadism may lead to incorrect therapeutic decisions, which may result in serious consequences for patients in the form of infertility or, in the case of athletes, the need to end their sports career due to problems with obtaining a TUE in the absence of the possibility of proving a permanent cause of hypogonadism. FSH and FSHR mutations or FSHR SNPs should always be suspected in the case of the coexistence of hypergonadotropic hypogonadism, low testicular volume, and reduced seminogram parameters after excluding Klinefelter syndrome and secondary testicular damage.

The greatest value of this clinical case lies in the observation that our treatment led to long-term maintenance of normal testosterone levels after discontinuation of gonadotropin therapy. Although the observation period is currently only a few months, monitored testosterone levels do not decrease and even tend to increase slightly. If the patient’s testosterone levels are persistently elevated, this indicates that the implemented therapy is restoring normal testicular function in patients with FSHR variants regarding testosterone production and may protect them from lifelong testosterone replacement therapy.

## Data Availability

The original contributions presented in the study are included in the article/supplementary material. Further inquiries can be directed to the corresponding author.
